# Managing executive dysfunction following acquired brain injury and stroke using an ecologically valid rehabilitation approach: a study protocol for a randomized, controlled trial

**DOI:** 10.1186/1745-6215-14-306

**Published:** 2013-09-22

**Authors:** Deirdre R Dawson, Nicole D Anderson, Malcolm A Binns, Carolina Bottari, Thecla Damianakis, Anne Hunt, Helene J Polatajko, Merrick Zwarenstein

**Affiliations:** 1 Rotman Research Institute, Baycrest, 3560 Bathurst Street, Toronto, ON M6A 2E1, Canada; 2 Department of Occupational Science and Occupational Therapy, University of Toronto, Suite 160-500 University Avenue, Toronto, ON M5G 1V7, Canada; 3 Graduate Department of Rehabilitation Sciences, University of Toronto, Suite 160-500 University Avenue, Toronto, ON M5G 1V7, Canada; 4 Center for Interdisciplinary Research in Rehabilitation of Greater Montreal, 2275 avenue Laurier Est, Montreal, Quebec H2H 2N8, Canada; 5 School of Social Work, University of Windsor, 401 Sunset Ave, Windsor, ON N9B 3P4, Canada; 6 Kunin-Lunenfeld Applied and Evaluative Research Unit, Baycrest, 3560 Bathurst Street, Toronto, ON M6A 2E1, Canada; 7 Schulich School of Medicine and Dentistry, University of Western Ontario, 245-100 Collip Circle, Research Park, London, ON N6G 4X8, Canada

**Keywords:** Cognitive orientation to daily occupational performance, Executive dysfunction, Occupational therapy, Rehabilitation, Strategy-training, Stroke, Traumatic brain injury

## Abstract

**Background:**

We have been investigating an ecologically valid strategy-training approach to enable adults with executive dysfunction to attain everyday life goals. Here, we report the protocol of a randomized controlled trial of the effects of this training compared to conventional therapy in a sample of community-dwelling adults with acquired brain injury and/or stroke.

**Methods/design:**

We will recruit 100 community-dwelling survivors at least six months post-acquired brain injury or stroke who report executive dysfunction during a telephone interview, confirmed in pre-training testing. Following pre-training testing, participants will be randomized to the ecologically valid strategy training or conventional therapy and receive two one-hour sessions for eight weeks (maximum of 15 hours of therapy). Post-testing will occur immediately following the training and three months later. The primary outcome is self-reported change in performance on everyday life activities measured using the Canadian Occupational Performance Measure, a standardized, semi-structured interview. Secondary outcomes are objective measurement of performance change from videotapes of treatment session, Performance Quality Rating Scale; executive dysfunction symptoms, Behavioural Rating Inventory of Executive Function – Adult; participation in everyday life, Mayo-Portland Adaptability Inventory Participation Index; and ability to solve novel problems, Instrumental Activities of Daily Living Profile.

**Discussion:**

This study is of a novel approach to promoting improvements in attainment of everyday life goals through managing executive dysfunction using an ecologically valid strategy training approach, the Cognitive Orientation to daily Occupational Performance. This study compares the efficacy of this approach with that of conventional therapy. The approach has the potential to be a valuable treatment for people with chronic acquired brain injury and/or stroke.

**Trial registration:**

clinicaltrials.gov, Trial Identification Number: NCT01414348

## Background

Executive processes, that is high-level cognitive functions involved in control and direction of action, including planning, monitoring, initiating, and switching, have been described by Lezak as being the heart of all socially useful, personally enhancing, constructive and creative activities [1]. Thus, impairments of executive function can have the most devastating impact on everyday life because of their super ordinate role in behavioural and cognitive processing [2]. Considerable attention has been directed at developing and testing interventions to manage these impairments; in the past six years, four systematic reviews and one meta-analysis related to treatment for executive dysfunction have been published [3]– [6].

The majority of studies in this area use some form of problem-solving or metacognitive strategy instruction. Meta-cognitive strategy instruction has roots in cognitive-, educational- and neuro-psychology [7]– [9]. Luria, in his seminal work, was perhaps the first to hypothesize explicit steps in the problem-solving process: problem representation, planning, execution and evaluation [8]. Across the work done in this area, different aspects of the process have been emphasized and, in some cases, additional steps have been included. For example, Levine et al., in a study using metacognitive strategy instruction with adults with traumatic brain injury, emphasize stopping (‘defined as periodic suspension of ongoing activities to assess goal attainment’ , p. 145) as critical for preventing absent-minded slips which would interfere with defining the problem at hand [10]. In contrast, Rath et al. focused on problem orientation, defined as focusing on removing impediments to effective use of problem-solving skills (i.e., cognitive distortions, misattributions, illogical thinking) to promote effective use of problem-solving skills [11]. Spikman et al. built on Levine’s work but placed a broader emphasis on self-awareness so that throughout their program participants were asked to predict and then evaluate their successes and/or failures [12]. In addition, the control condition varied across these studies with only one using conventional therapy, although this study did not report whether they controlled for time spent in therapy, the experimental condition was provided by trained neuropsychologists and the control condition consisted of ‘usual therapy, such as physiotherapy’ (p. 525) [13]. Despite the differences, as a whole, these studies provide compelling evidence that problem-solving or meta-cognitive training result in positive post-intervention changes including, in some instances, improvements in untrained everyday life activities, suggesting that the training has resulted in participants’ ability to transfer what they have learned to novel, untrained activities [11]– [14].

Our pilot work on an ecologically valid approach to strategy-training, an adapted version of the Cognitive Orientation to daily Occupational Performance (CO-OP) approach, also showed changes in everyday life on untrained activities that were maintained at three-month follow-up [15,16]. The CO-OP approach reflects the understanding of real-world participation posited by the World Health Organization in the International Classification of Functioning [17], that participation in the real world results from a complex interaction between impairments in bodily structures and functions (e.g., brain pathology), the person’s attributes (e.g., education, intelligence), and the environment within which they are functioning. To be maximally effective, a rehabilitation intervention must address each of these. The CO-OP approach does this by including an explicit metacognitive strategy at its core: this rubric (stated simply as Goal, Plan, Do, Check) is taught to and practiced with patients in the context of their self-identified difficulties in real-world activities and in the environment in which these difficulties occur [18]. Thus, this approach has maximum ecological validity: rather than simulating real-world activity, the adapted CO-OP approach takes place in the real world, with all of its complexities and demands. Further, it is well suited to reflecting the current health care focus of maintaining people in the community.

The CO-OP approach, like many other rehabilitation interventions, can be considered a complex intervention in that it involves a number of interacting components [19]– [21]. A key question identified in relation to evaluating complex interventions is, does the intervention work in everyday practice [20]? To address this question, we have designed a two-group study for people with acquired brain injury and stroke; the experimental group will receive CO-OP while the control group will receive conventional rehabilitation. Both interventions will be delivered in the community.

### Objective

To discover whether the novel intervention, the adapted CO-OP approach, is more effective than conventional occupational therapy for (a) improving ability to achieve self-identified goals in everyday life; and (b) achieving far transfer demonstrated as overall reduced impact of executive dysfunction in everyday life and overall improved community integration.

## Methods/design

### Design

This study will examine the effects of the adapted form of the CO-OP approach in community-dwelling adults with executive dysfunction arising from acquired brain injury or stroke. Following a telephone-screening interview, participants will attend a baseline testing session and be randomized to the experimental or control interventions. This is a masked trial: participants are told they are receiving one of two forms of rehabilitation therapy both of which will address daily living activities that are difficult to perform. Training, over a 10-week period (up to 15 one-hour sessions), will be followed by post-training and follow-up (3-months) testing.

### Participants and recruitment

**Figure 1:**
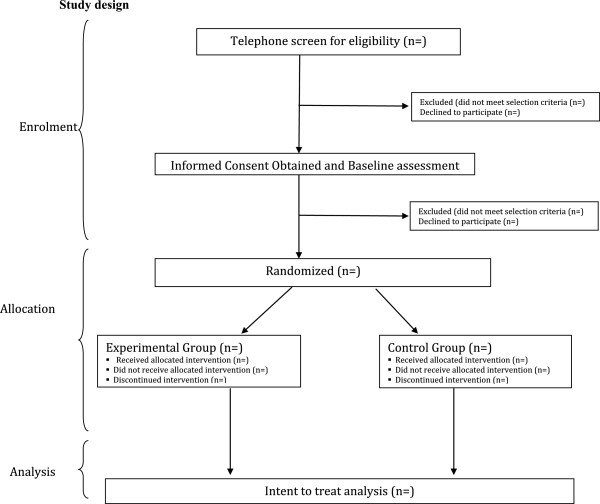
CONSORT flow diagram.

One hundred community-dwelling (within the Greater Toronto area) survivors of acquired brain injury or stroke will be recruited (Figure 1, CONSORT diagram). Participants will be recruited via (1) flyers distributed to a broad network of community agencies providing services to persons with stroke and brain injury; (2) flyers posted on brain injury society and agency websites (e.g., Acquired Brain Injury Network of the Greater Toronto Area); (3) through the Heart and Stroke Foundation Centre for Stroke Recovery research subject pool; and (4) word of mouth. Respondents will be screened via a telephone interview to determine eligibility. Inclusion criteria for the trial are age 18 years or greater; fluent in written and spoken English; have sustained a traumatic brain injury, another form of acquired brain injury that is not related to a congenital, developmental or degenerative disorder but which occurred through a medical problem or disease process (e.g., anoxia, meningitis, subarachnoid haemorrhage, non-malignant tumour removal) and/or cerebrovascular accident; and have evidence of exe-cutive dysfunction on neuropsychological and behavioural testing. Participants must also be able to self-identify areas of day-to-day difficulties that they would like to improve.

Exclusion criteria include other significant neurological or psychiatric history (e.g., multiple sclerosis, psychiatric illness requiring hospitalization), concurrent depression (measured using the Centre for Epidemiological Studies Depression Scale) [22], and/or substance abuse and concurrent receipt of occupational (or similar) therapy services.

We will also recruit a significant other for each participant to answer questions about the impact of executive function impairments on their loved one’s day-to-day life at pre-training, post-training, and follow-up testing.

### Ethical considerations and informed consent

Ethics approval has been received from the Baycrest Research Ethics Board. A trained research assistant will explain the purpose of the study and potential risks to potential participants. Participants will receive an information letter about the study and there will be no time limit for them to consider implications, ask questions, and respond to the invitation to participate. Participants will be informed that they are free to withdraw from the treatment under study at any time for any reason although they will be invited to come back for post-training and follow-up assessments.

### Sample size determination

Our preliminary data with adults with traumatic brain injury divided into a treatment (n = 6) and an inactive comparison group (n = 6) yielded a very large effect size of *d* (standardized difference of means) = 1.22 on self-rated changes in performance on the Canadian Occupational Performance Measure [23]. The comparison group showed similar performance scores prior to and following the control period (average increase 0.27 ± 0.98) while the intervention group showed an increase between pre-training and post-training testing sessions (average increase 1.69 ± 1.33). We powered this study to detect a moderate effect size as we are comparing the experimental treatment to conventional therapy (as opposed to a no-treatment control group). We are also including a third test session for longer-term follow-up. To achieve 80% power to detect a moderate effect size (*d* = 0.6) with alpha at 5% we need 45 participants per group [24]. We are over sampling by 10% to account for potential attrition. Thus, we will recruit 50 people for each arm of the study.

### Randomization

Randomization will be done in blocks of four via a random numbers table. Following baseline testing and participant registration, the Research Assistant will open the next sealed opaque envelope.

### Measures

All participants will be characterized using socio-demographic (age, gender, education, estimated pre-morbid IQ), event-related (time post-injury/stroke, lesion burden), and neuropsychological status (attention, memory, executive function) variables. Pre-morbid IQ will be estimated using the vocabulary subtest from the WAIS-III [25]. The neuropsychological test battery will be comprised of tests of (1) attention (Symbol Digit Modalities Test) [26], (2) memory (Hopkins Verbal Learning Test) [27], and executive function (Delis-Kaplan Executive Function System, Trail Making, Tower and Verbal Fluency Tests) [28].

### Primary outcome measure

The primary outcome measure is the Canadian Occupational Performance Measure (COPM), a standardized, semi-structured interview [23], selected as it has been used as the primary outcome measure in previous studies on the CO-OP approach and in other intervention studies with people with acquired brain injury as well as other populations [29]– [31]. The COPM interview results in participants identifying specific everyday activities of daily living (including leisure and work-related activities) that they need to, want to, and/or are expected to do, but are having difficulty with. In this study, the COPM will be administered by a trained occupational therapist as part of the pre-training assessment. During the interview, participants will identify five to six areas of everyday life that are problematic and then state these as goals. For example, if someone identifies meal preparation as a problem area, the goal statement might be ‘to prepare dinner for my family three nights each week’. Participants will rate their current performance of each goal on a 10-point Likert type scale (1 = not able to do it all, 10 = able to extremely well).

Prior to the beginning of the training, participants will be asked to select one of the goals for training and the Research Assistant will randomly select two additional goals (of the remaining four or five) for training; thus, three goals will be trained. The remaining goals will not be trained. After goals are identified by participants, their significant other will independently rate their perception of their family member’s performance. The primary outcome is the participants’ self-reported rating of performance for all untrained goals. This score serves as a measure of transfer of training. At post-test and follow-up, participants and their significant others will rate their performance without reference to the ratings done previously.

### Secondary outcome measures

We will objectively measure performance change during treatment sessions using the Performance Quality Rating Scale (PQRS), a 10-point observation scale (1 = not able to do it all; 10 = being able to do the specified activity with good quality) [18]. Videotapes of treatment sessions will be scored by trained occupational therapists blind to the study arm and session time point. The PQRS has been used in many of the previous studies on the CO-OP intervention [32,33], with reported reliability of 0.72 [34]. We will further establish inter-rater reliability using videotapes from our pilot work. Intra-class correlation coefficients will be calculated and a coefficient of 0.7 will be considered sufficient [32].

Three additional outcome measures will be used to determine the benefits of the experimental intervention on the perceived and observed impact of the executive dysfunction in everyday life and overall improved community integration. The Behavioural Rating Inventory of Executive Function – Adult is a standardized 75-item questionnaire that asks persons to rate the frequency with which particular aspects of the dysexecutive syndrome occur in their day-to-day lives, including emotional or personality changes, motivation changes, behavioural changes, and cognitive changes [35]. This will be administered to both the participant and their significant other.

As improving performance on meaningful everyday life activities should ultimately have a positive influence on measures of community integration or community partici-pation, we will measure changes in community integration using the Mayo-Portland Adaptability Inventory Participation Index [36], an 8-item questionnaire that includes items on financial management, work and school, social relationships and leisure. We will use both self and significant other versions.

The ability of participants to solve novel performance problems will be measured using two sub-scales of the Instrumental Activities of Daily Living Profile [33], namely obtaining information and making a budget. In the first task, participants have to obtain specific information about how to get from one large urban area to another by a specified mode of transportation and in the second they have to prepare an annual budget within specific task constraints (e.g., income level, have to save for car). Both tasks will be videotaped and performance scored by a rater blinded to group and session on a three-point scale (independent, independent with difficulty, unable to complete task). Time to completion will also be recorded.

### Intervention

#### Experimental intervention

The adapted CO-OP intervention involves up to 15 hours of treatment delivered by a trained occupational therapist. Key elements of the approach are that: i. Participants are actively engaged in selecting their own treatment goals. ii. A meta-cognitive strategy is used (goal-plan-do-check) throughout each session to promote goal achievement. Participants are trained to use the meta-cognitive strategy as a way of planning and regulating their performance. Workbooks are provided to participants so that they, with the assistance of the clinician, can keep track of individual goals and plans. iii. During each intervention session, participants are ‘guided’ by the therapist to use the meta-cognitive strategy to identify their performance problem (i.e., dynamic performance analysis) and discover the plans or strategies that will solve these problems. ‘Guided discovery’ is a learning concept rooted in general principles of learning theory [37]. There is evidence that the participant’s work of discovering the ‘solution’ or strategies is integral to the success of the intervention and contributes to the development of self-efficacy as participants can attribute their successes to their own plans [38]. Guided discovery means that the therapist takes a non-directive approach; they can offer suggestions that point participants to feasible plans although these suggestions are not mandated. Participants decide which plans to ‘do’ , check to see which ones work and through the course of the intervention iteratively test a number of plans (plan-do-check) and learn that not all plans work; thus, they learn to self-monitor and correct their performance. iv. A focus in each session is on training participants to generalize and transfer their learning. This is done at each session by explicitly discussing with participants how else they have or could have used the identified strategies and how they will continue to use them. The combination of these features is unique and novel. To our knowledge, no other intervention utilizes all of these features and we believe this approach represents a major step forward in rehabilitation.

#### Control intervention – conventional occupational therapy

Participants in the control intervention will receive up to 15 hours of therapy from a trained occupational therapist provided with a similar ‘referral’ as to what might be received by a publically-funded community therapist in Ontario, Canada, namely age of participant, diagnosis, and reason for referral (in this instance the participant-identified difficulties in everyday life). The content of therapy will include one or more of the following: task-specific training in activities of daily living; environmental and task modifications; and provision and training in the use of compensatory memory devices [39]. Although there are advantages and disadvantages to every control intervention, conventional therapy was chosen as it is highly acceptable to participants and therapists, maximizes equipoise, and has distinct benefits for knowledge exchange as the external validity (generalizability of the results) is maximized [40,41].

### Planned analyses

Differences in participants’ baseline socio-demographic, injury related, and neuropsychological status will be characterized by using proportion for gender and mean and standard deviations for other variables. To address the primary objective regarding efficacy of the experimental treatment, the main analysis will be performed as follows. The mean and standard deviation of the primary outcome (self-rating of performance on untrained goals) will be reported for both study groups. An analysis of covariance model for performance rating at each of post-treatment and follow-up time points will be fit to examine the effect of intervention group. Baseline performance rating and treatment intensity (measured in hours) will be included in the model as linear, continuous covariates. The contrast between groups adjusted for baseline and treatment intensity will be reported along with 95% confidence interval. Secondary outcomes will be reported in a similar fashion.

## Discussion

This paper describes the protocol for testing the efficacy of an ecologically valid approach to managing executive dysfunction. We hypothesize that participants trained in the adapted CO-OP approach and their significant others will report and demonstrate significantly more improvement on self-identified difficulties in everyday life than participants receiving conventional therapy. We also hypothesize that the strategies learned by participants receiving the CO-OP training will be applied in other areas of everyday life, will be reported by participants and their significant others and observed on measures of the impact of executive dysfunction in everyday life and on overall community integration.

Comparing the CO-OP approach to conventional occupational therapy will ultimately allow us to make recommendations about the treatment of people with stroke and acquired brain injury.

## Trial status

This article was submitted on July 4, 2013. To date, 42 participants have been enrolled.

## Abbreviations

CO-OP: Cognitive orientation to daily occupational performance; COPM: Canadian occupational performance measure; PQRS: Performance quality rating scale.

## Competing interests

H. Polatajko receives royalties from the published manual on the CO-OP approach and fees for workshops for teaching this approach. All other others declare that they have no competing interests.

## Authors’ contributions

DD, NA, MB, CB, TD, HP, and MZ participated in the conception and design of the trial and in plans for the analysis of the data. DD, HP, and AH participated in the design of the experimental intervention. DD drafted the manuscript. All authors commented critically on the manuscript and approved the protocol for publication.

## References

[B1] Lezak MD The problem of assessing executive functions Intl J Psych 1982 17 281 297 10.1080/00207598208247445

[B2] Cicerone K Levin H Malec J Stuss D Whyte J Cognitive rehabilitation interventions for executive function: moving from bench to bedside in patients with traumatic brain injury J Cog Neurosci 2006 18 1212 1222 10.1162/jocn.2006.18.7.1212 16839293

[B3] Kennedy MRT Coelho C Turkstra L Ylvisaker M Sohlberg MM Yorkston K Chiou HH Kan PF Intervention for executive functions after traumatic brain injury: a systematic review, meta-analysis and clinical recommendations Neuropsychol Rehabil 2008 18 257 299 10.1080/09602010701748644 18569745

[B4] Rohling M Faust M Beverly B Demakis G Effectiveness of cognitive rehabilitation following acquired brain injury: a meta-analytic re-examination of cicerone et al.’s (2000, 2005) Systematic reviews Neuropsychol 2009 23 20 39 10.1037/a0013659 19210030

[B5] Cicerone KD Langenbahn DM Braden C Malec JF Kalmar K Fraas M Felicetti T Laatsch L Harley JP Bergquist T Azulay J Cantor J Ashman T Evidence based cognitive rehabilitation: updated review of the literature from 2003 through 2008 Arch Phys Med Rehabil 2011 92 519 530 10.1016/j.apmr.2010.11.015 21440699

[B6] Chung CSY Pollock A Campbell T Durward BR Hagen S Cognitive rehabilitation for executive dysfunction in adults with stroke or other adult non-progressive acquired brain damage Cochrane Database Syst Rev 2013 4 1 76 10.1002/14651858.CD008391.pub2 PMC6464714 23633354

[B7] Fox E Riconscente M Metacognition and self-regulation in James, Piaget & Vygotsky Educ Psychol Rev 2008 20 373 389 10.1007/s10648-008-9079-2

[B8] Luria AR Higher Cortical Functions in Man 1980 2 New York: Basic Books

[B9] Meichenbaum D Cognitive-Behaviour Modification: An Integrative Approach 1977 New York: Plenum Press

[B10] Levine B Stuss DT Winocur G Binns MA Fahy L Mandic M Bridges K Robertson IH Cognitive rehabilitation of the elderly: effects on strategic behavior in relation to goal J Int Neuropsych Soc 2007 13 143 152 10.1017/S1355617707070178 17166313

[B11] Rath JF Simon D Langenbahn DM Sherr RL Diller L Group treatment of problem-solving deficits in outpatients with traumatic brain injury: a randomised outcome study Neuropsychol Rehabil 2003 13 461 488 10.1080/09602010343000039

[B12] Spikman JM Boelen DHE Lamberts KF Brouwer WH Fasotti L Effects of a multifaceted program for executive dysfunction after acquired brain injury on indications of executive functioning in daily life J Int Neuropsych Soc 2010 16 118 129 10.1017/S1355617709991020 19900348

[B13] Miotta EC Evans JJ Souza de Lucia MC Scaff M Rehabilitation of executive dysfunction: a controlled trial of attention and problem solving treatment group Neuropsychol Rehabil 2009 19 517 540 10.1080/09602010802332108 18766984

[B14] Novakovic-Agopian T Chen AJ Rome S Abrams G Castelli H Rossi A McKim R Hills N D’Esposito M Rehabilitation of executive functioning with training in attention regulation applied to individually defined goals: a pilot study bridging theory, assessment, and treatment J Head Trauma Rehabil 2011 26 325 328 10.1097/HTR.0b013e3181f1ead2 21169860

[B15] Dawson DR Gaya A Hunt A Lemsky C Levine B Lo A Polatajko H Using the cognitive orientation to occupational performance with adults with traumatic brain injury Can J Occupl Ther 2009 76 115 127 10.1177/000841740907600209 19456090

[B16] Dawson DR Binns M Hunt A Lemsky C Polatajko HJ Occupation based strategy training for adults with traumatic brain injury: a pilot study Arch Phys Med Rehabil 2013 94 1959 63 10.1016/j.apmr.2013.05.021 23796683

[B17] World Health Organization International Classification of Functioning, Disability and Health 2001 Geneva: WHO

[B18] Polatajko H Mandich A Enabling Occupation in Children: The Cognitive Orientation to Daily Occupational Performance (CO-OP) Approach 2004 Ottawa: CAOT Pub

[B19] Campbell M Fitzpatrick R Haines A Kinmonth AL Sandercock P Spiegelhalter D Tyrer P Framework for design and evaluation of complex interventions to improve health Brit Med J 2000 321 694 696 10.1136/bmj.321.7262.694 10987780 PMC1118564

[B20] Craig P Dieppe P Macintyre S Mitchie S Nazareth I Petticrew M Developing and evaluating complex interventions: the new medical research council guidance BMJ 2008 337 979 983 10.1136/bmj.a979 PMC2769032 18824488

[B21] Hart T Treatment definition in complex rehabilitation interventions Neuropsychol Rehabil 2009 19 824 840 10.1080/09602010902995945 19544183

[B22] Radloff LS The CES-D Scale: a self-report depression scale for research in the general population Appl Psychol Meas 1977 1 385 401 10.1177/014662167700100306

[B23] Law M Baptiste S McColl MA Opzoomer A Polatajko H Pollock N Canadian Occupational Performance Measure 1998 3 Ottawa: CAOT Publications

[B24] Cohen J Statistical Power Analysis for the Behavioral Sciences 1988 2 Hillsdale, NJ: Lawrence Earlbaum Associates

[B25] Wechsler D The Wechsler Adult Intelligence Scale – Revised 1985 New York: The Psychological Corporation

[B26] Smith A Symbol Digit Modalities Test 1978 Los Angeles: Western Psychological Services

[B27] Brandt J The Hopkins verbal learning test: development of a new memory test with six equivalent forms Clin Neuropsychol 1991 5 125 142 10.1080/13854049108403297

[B28] Delis D Kaplan E Kramer J Delis-Kaplan Executive Function System 2001 San Antonio, TX: The Psychological Corporation

[B29] Clare L Linden DEJ Woods RT Whitaker R Evans SJ Parkinson C van Paasschen J Nelis SM Hoare Z Yuen KSL Rugg MD Goal-oriented cognitive rehabilitation for people with early-stage Alzheimer disease: a single-blind randomized controlled trial of clinical efficacy Am J Geriat Psychiat 2010 18 928 939 10.1097/JGP.0b013e3181d5792a 20808145

[B30] McEwen S Polatajko HJ Huijbregts MPJ Ryan JD Exploring a cognitive-based treatment approach to improve motor-based skill performance in chronic stroke: results of three single-case experiments Brain Inj 2009 23 1041 1053 10.3109/02699050903421107 19909052

[B31] Sewell L Singh SJ Williams JEA Collier R Morgan MDL Can individualized rehabilitation improve functional independence in elderly patients with COPD? Chest 2005 128 1194 1200 10.1378/chest.128.3.1194 16162706

[B32] Nunnally J Psychometric Theory 1978 New York: McGraw-Hill

[B33] Bottari C Dassa C Rainville CM Dutil E The IADL Profile: development, content validity, intra- and interrater agreement Can J Occup Ther 2010 77 90 100 10.2182/cjot.2010.77.2.5 20464894

[B34] Martini R Verbal Self-guidance as an Approach to the Intervention of Children with Developmental Coordination Disorder: A Systematic Replication Study Master’s thesis: University of Western Ontario, School of Occupational Therapy 1994

[B35] Roth RM Peter KI Gioia GA Behaviour Rating Inventory of Executive Function – Adult Version 2005 Lutz, FL: Psychological Assessment Resources Inc.

[B36] Malec J Lezak M Manual for the mayo-Portland adaptability inventory (MPAI-4) http://tbims.org/combi/mpai/manual.pdf

[B37] Mayer R Should there be a three-strikes rule against pure discovery learning? The case for guided methods of instruction Am Psychol 2004 59 14 19 14736316 10.1037/0003-066X.59.1.14

[B38] Trombly C Radomski M Trexel C Burnet-Smith S Occupational therapy and achievement of self-identified goals by adults with acquired brain injury: phase II Am J Occup Ther 2002 56 489 498 10.5014/ajot.56.5.489 12269503

[B39] Gillen G Cognitive and Perceptual Rehabilitation: Optimizing Function 2009 St. Louis, MO: Mosby

[B40] Hart T Fann JR Novack TA The dilemma of the control condition in experience-based cognitive and behavioural treatment research Neuropsych Rehabil 2008 18 1 21 10.1080/09602010601082359 17852761

[B41] Zwarenstein M Treweek S What kind of randomized trials do we need? Can Med Assoc J 2009 180 998 1000 10.1503/cmaj.082007 19372438 PMC2679816

